# Vestibular and balance function in veterans with chronic dizziness associated with mild traumatic brain injury and blast exposure

**DOI:** 10.3389/fneur.2022.930389

**Published:** 2022-09-01

**Authors:** Faith W. Akin, Owen D. Murnane, Courtney D. Hall, Kristal M. Riska, Jennifer Sears

**Affiliations:** ^1^Mountain Home Hearing and Balance Research Program, James H. Quillen VA Medical Center, Mountain Home, TN, United States; ^2^Department of Audiology and Speech-Language Pathology, East Tennessee State University, Johnson City, TN, United States; ^3^Physical Therapy Program, Department of Rehabilitative Sciences, East Tennessee State University, Johnson City, TN, United States; ^4^Department of Head and Neck Surgery & Communication Sciences, Duke University School of Medicine, Durham, NC, United States

**Keywords:** vestibular diseases, vestibular function tests, utricle and saccule, postural balance, traumatic brain injury, blast (explosion) wave-induced neurotrauma

## Abstract

The purpose of this study was to examine vestibular and balance function in individuals with chronic dizziness associated with mTBI/blast. A prospective case-control study design was used to examine ocular motor, vestibular function, and postural stability in veterans with symptoms of dizziness and/or imbalance following an mTBI or blast exposure (*n* = 77) and a healthy control group (*n* = 32). Significant group differences were observed for saccadic accuracy, VOR gain during slow harmonic acceleration at 0.01 Hz, cervical vestibular evoked myogenic potentials asymmetry ratio, composite equilibrium score on the sensory organization test, total Dynamic Gait Index score, and gait. The frequency of test abnormalities in participants with mTBI/blast ranged from 0 to 70% across vestibular, ocular motor, and balance/gait testing, with the most frequent abnormalities occurring on tests of balance and gait function. Seventy-two percent of the mTBI/blast participants had abnormal findings on one or more of the balance and gait tests. Vestibular test abnormalities occurred in ~34% of the individuals with chronic dizziness and mTBI/blast, and abnormalities occurred more frequently for measures of otolith organ function (25% for cVEMP and 18% for oVEMP) than for measures of hSCC function (8% for SHA and 6% for caloric test). Abnormal ocular motor function occurred in 18% of the mTBI/blast group. These findings support the need for comprehensive vestibular and balance assessment in individuals with dizziness following mTBI/blast-related injury.

## Introduction

Dizziness is one of the more common and persistent symptoms following a head injury or concussion (1, 2). In war-related injuries, mild traumatic brain injury (mTBI) is often associated with a blast exposure, and the same insult that produces TBI can cause trauma to the inner ear. The impact of blast on the auditory system is well established (3), however, there is less known about the impact of blast on the vestibular organs. Dizziness and imbalance can also occur following blast exposure (4, 5), and damage to the vestibular sensory organs has been described in blast victims (6). Most studies have focused on the effect of head trauma or TBI, and less is known about the effect of blast exposure on peripheral vestibular function.

For many individuals with blast-related mTBI, the cause(s) of their dizziness or imbalance is unclear as many studies limit the evaluation of vestibular and balance function to a symptom-based questionnaire [e.g., (7, 8)]. A limitation of this method is that non-vestibular disorders may cause dizziness or imbalance, and many individuals who complain of dizziness or imbalance have normal vestibular function. Another shortcoming is that many studies on dizziness (particularly studies of sports-related concussion) have limited their assessment to bedside screening and balance tests. Although loss of vestibular function may result in postural instability, postural stability involves the dynamic interplay between multiple body systems, including the sensory, central nervous and musculoskeletal systems. Abnormal balance function, therefore, may not be a sensitive clinical indicator of vestibular dysfunction (9).

It is well established that head injury can result in peripheral vestibular hypofunction (or unilateral weakness on the caloric test) (10), and it is reasonable to presume that peripheral vestibular system abnormalities associated with TBI are likely due to the head trauma rather than the resulting brain injury. Several studies have examined vestibular function in individuals with post-concussive dizziness and blast exposure by measuring the vestibulo-ocular reflex (VOR)/horizontal semicircular canal (hSCC) response to caloric irrigation, and most studies demonstrated that hSCC dysfunction occurs in less than a quarter of individuals with dizziness following head injury (11, 12). Until recently, the literature on vestibular consequences of head injury was restricted to the inner ear vestibular assessment of horizontal semicircular canal function and its connections to the eyes (VOR). Peripheral vestibular loss, however, can occur in one or both labyrinths, in one or both branches of the vestibular nerve, and in one or more vestibular sensory organs. Cervical vestibular evoked myogenic potentials (cVEMP) assess the sacculo-collic pathway and have been used in the past decade to determine the impact of TBI on otolith organ function. Similarly, ocular VEMPs (oVEMPs) can be used to measure utricular pathway function. There is emerging evidence in animals and humans that otolith organ dysfunction may occur more often than horizontal canal (VOR) dysfunction in individuals with dizziness following mTBI or blast exposure and the saccule may be particularly susceptible to blast-related damage and noise exposure due to the anatomic proximity of the saccule to the stapes footplate (13–18).

The purpose of this study was to examine vestibular and balance function in individuals with chronic dizziness associated with mTBI/blast. We hypothesized that individuals with mTBI/blast and dizziness will have decreased vestibular function and postural stability compared to healthy controls. We also hypothesized that the sacullo-collic pathway (cVEMPs) is particularly susceptible to mTBI/blast compared to the horizontal canal/VOR pathway.

## Materials and methods

### Participants

A prospective case-control study design was used to examine the effects of blast and/or mTBI on the vestibular system and postural stability. Two groups were enrolled in the study: (1) veterans with symptoms of dizziness and/or imbalance following a blast exposure and/or mTBI (mTBI/blast; *n* = 77) and (2) a control group comprised of individuals (veterans and non-veterans) with no complaints of dizziness or imbalance and no self-reported history of TBI or blast exposure (Control; *n* = 32). Participants were recruited from the Polytrauma and Audiology clinics at the Mountain Home Veterans Affairs (VA) Medical Center and from the local university, medical school, and community. A history of mTBI was determined by a physician diagnosis in the VA computerized patient record system (CPRS). The VA CPRS was also used to record a history of post-traumatic stress disorder (PTSD) for the veteran participants. A history of blast exposure was documented using the Walter Reed Army Medical Center Blast Injury Questionnaire (19). Exclusion criteria included a prior history of vestibular or neurological disorders, lower extremity joint replacement or amputation, cognitive impairment, and best-corrected visual acuity worse than 20/40 in the better eye (20) because these factors can impact postural control independent of vestibular function. All participants were screened for appropriate cognitive function based on age and education (21) *via* the Mini-Mental State Exam (22) using a score of ≥24 required for inclusion to ensure sufficient cognitive function to complete the vestibular and balance assessment.

This study was approved by the East Tennessee State University/James H. Quillen VA Medical Center Institutional Review Board. All participants completed a written informed consent form prior to participation in the study and were given nominal payment for their time.

### Protocol

Each participant underwent comprehensive vestibular and balance assessment. Participants were asked to refrain from the use of alcohol, recreational drugs, over-the-counter antihistamines, anti-dizzy medications, and sleeping pills for 48 h prior to testing. Laboratory vestibular assessment included tests of ocular motor, horizontal semicircular canal, and otolith organ function. Ocular motor tests (gaze evoked, smooth pursuit and saccades) were performed as part of the vestibular test battery to rule out central involvement. Caloric irrigation and slow harmonic acceleration in a rotary chair were performed to determine the effect of mTBI/blast exposure on hSCC/VOR function. Cervical and ocular vestibular evoked myogenic potentials (VEMPs) were measured to determine the effects of mTBI/blast exposure on otolith organ function. In addition, the Dix-Hallpike test and roll tests were performed to identify the presence of benign paroxysmal positional vertigo (BPPV). To examine the functional impact of mTBI/blast exposure on postural control, the sensory organization test and gait assessments were performed. Participants in the mTBI/blast group were asked to complete the Dizziness Handicap Inventory as a measure of self-perceived balance handicap (23). Behavioral audiometric assessment was performed prior to the vestibular assessment. Air- and bone-conduction pure-tone audiometry was performed, and clinical immittance testing was used to measure middle ear function (226-Hz tympanometry and ipsilateral acoustic reflex thresholds at 0.5, 1, and 2 kHz).

#### Vestibular tests

The Dix-Hallpike and roll tests were used to determine the presence or absence of BPPV. The maneuvers were performed in the head hanging left and head hanging right positions, and eye movement was recorded with video-oculography (RealEyes Monocular, Micromedical Technologies, Chatham IL). Abnormal findings were defined as the presence of brief nystagmus and vertigo in the provoking position.

Ocular motor testing included gaze testing, sinusoidal smooth pursuit, and random saccades, and was performed using video-oculography (System 2000, Micromedical Technologies, Chatham IL) to exam central pathways. Eye movement recording was calibrated prior to ocular motor testing. Gaze testing identified the presence or absence of pathologic gaze-evoked nystagmus with changes in gaze position (center, 20° right and left, 20° up and down). For sinusoidal smooth pursuit, gain was calculated as participants tracked a target at 0.1, 0.2, 0.4 Hz, and abnormal gain at 0.01 Hz was defined as <0.66 for ages 20–59 years and <0.53 for ages ≥60 years (Micromedical Technologies). The saccadic test paradigm used leftward and rightward targets presented randomly from 5 to 25°. The mean accuracy and latency were calculated across all leftward and rightward saccadic eye displacements for each participant. Abnormal saccadic accuracy was defined as <77 or >137%; abnormal saccadic latency was defined as >260 ms (Micromedical Technologies). Saccadic velocity data were only analyzed for participants who demonstrated accurate leftward and/or rightward eye displacements for 18 to 25° target displacements, and abnormal velocity was defined as <292 deg/s (24).

Caloric testing was performed using a computer-based videonystagmography (VNG) system (ICS Chartr, GN Otometrics, Schaumburg IL) to assess the hSCC/VOR pathway, and eye movement data were recorded, digitized, and analyzed. A water irrigator (ICS NCI-440, GN Otometrics, Schaumburg IL) was used to deliver the caloric stimulus; caloric irrigations consisted of 250 ml of water for 30 s at temperatures of 44 and/or 30°C with the participant in a supine position and the head elevated 30°. Following cessation of the caloric irrigations, each participant engaged in mental alerting tasks to avoid response suppression of the induced nystagmus. The peak of the response was calculated as the average slow component eye velocity (SCEV) of the three strongest beats of nystagmus. Normal responses were defined as either an inter-ear difference of ≤10% for the monothermal warm caloric test (25) or a unilateral weakness of ≤25% for the alternating binaural bithermal caloric test (26). Bithermal caloric irrigation was used if the monothermal warm inter-ear difference was >10%. Abnormal calorics were defined as a unilateral weakness >25% or a bilateral caloric weakness [total warm SCEV <11°/s and total cool SCEV <11°/s; (27)].

The rotary chair test (System 2000 Rotational Vestibular Chair, Micromedical Technologies, Chatham IL) and video-oculography was used to assess the hSCCs/SVN pathway during slow harmonic acceleration (SHA) over a frequency range that included 0.01, 0.04, and 0.16 Hz. Participants were seated in a light-proof booth with the head upright so that yaw-axis rotation occurred in the plane of both hSCCs, and mental alerting tasks were used to prevent suppression of the VOR response. Phase and gain were calculated for the SCEV response at each frequency. SCEV responses were defined as abnormal if gain at 0.01 was <0.25 and/or phase at 0.01 was >56 degrees (28). To examine visual fixation suppression (VFx) of the VOR, gain was measured with a fixation target during SHA at 0.16 Hz. Abnormal VFx was defined as gain >0.13 (Micromedical Technologies).

Cervical VEMPs were performed as a measure of saccular/inferior vestibular nerve (IVN) function [e.g., (29)]. Participants were seated upright and instructed to turn their head laterally to maximally contract the sternocleidomastoid (SCM) muscle. A two-channel recording of the cVEMP was obtained using the ICS Chartr^®^ EP200 (version 6.2.1). Non-inverting electrodes were placed at the midpoints of the SCM muscles, inverting electrode at the sternoclavicular junctions, and the ground electrode was placed on the forehead. Air conduction cVEMPs were recorded using 500-Hz tone-burst stimuli presented monaurally *via* insert earphones (Etymotic ER3A) at 120 dB _peak_SPL. If cVEMPs were absent at 120 dB _peak_SPL, then recordings were also obtained at 125 dB _peak_SPL. Bone conduction cVEMPs were obtained in participants with absent AC cVEMPs and evidence of middle ear pathology. The magnitude of the tonic EMG level was recorded from the non-inverting electrode and obtained simultaneously in a third channel during the cVEMP recording, and the cVEMP amplitude was normalized for EMG level. cVEMPs were also obtained at 94 dB _peak_SPL to screen for superior semicircular canal dehiscence (SSCD).

The following measurements were calculated for each participant and compared across groups: (1) peak-to-peak cVEMP amplitudes (P1-N1) at 120 dB _peak_SPL, (2) P1 and N1 latencies at 120 dB _peak_SPL, and (3) asymmetry ratios. Asymmetry ratios (AR) were calculated from normalized P1-N1 amplitudes at 120 or 125 dB _peak_SPL using the following formula:


$$\begin{array}{l} AR=\;\frac{\left|left\;side\;P1-N1\;-\;right\;side\;P1-N1\right|}{\left|left\;side\;P1-N1\;\pm \;right\;side\;P1-N1\right|}\;x\;100 \end{array}$$

The ARs range from 100% to 0% with values near 0% indicating that P1-N1 amplitudes are symmetrical. The criterion for abnormal cVEMP was defined as an absent cVEMP at 125 dB _peak_SPL and/or a corrected cVEMP amplitude asymmetry ratio ≥40% or present cVEMP response at 94 dB _peak_SPL (laboratory normative data).

Ocular VEMPs (oVEMPs) were used as a measure of utricular and superior vestibular nerve (SVN) function (30). Recording electrodes were placed 1 cm below (non-inverting electrode) and 3 cm below (inverting electrode) the center of each pupil, and the ground electrode was at Fpz. Participants were seated in a reclining chair with their gaze fixed on a stationary target located one meter straight ahead at a vertical gaze angle of 30°. The stimulus was a 500-Hz Blackman windowed tone-burst (rarefaction onset phase; rise/fall time = 1 cycle and no plateau) presented at a repetition rate of 5 Hz and at a level of 142 dB peak Force Level (re: 1 μNewton; _peak_FL). The stimulus level was measured using an artificial mastoid (Bruel & Kjær, model 4930) and a sound-level meter (Bruel & Kjær, model 2250). Stimuli were generated by a commercial evoked potential instrument (ICS Chartr^®^ EP200; version 6.2.1), amplified (Bruel & Kjær power amplifier, model 2810; drive voltage of 5 V peak-to-peak), and delivered by a hand-held vibrator (Bruel & Kjær Mini-Shaker, model 4810) fitted with a custom acrylic rod that measured 9.2 cm in length and 2.5 cm in diameter. The Mini-Shaker was hand-held by the examiner such that the axis of the acrylic rod was approximately perpendicular to the subject's skull at a standard EEG electrode location (Fz). Prior to stimulation, the Fz location was marked on each subject's head according to the 10–20 electrode system of the International Federation (31). The weight of the Mini-Shaker (1 kg) was used to standardize the force of the shaker against the skull as no additional force was applied to Fz by the examiner. Abnormal oVEMP was defined as an absent response and/or an oVEMP amplitude asymmetry ratio ≥40% (laboratory normative data).

The following measurements were calculated for each participant and compared across groups: (1) peak-to-peak oVEMP amplitudes (N1-P1), (2) N1 and P1 latencies, and (3) asymmetry ratios. Asymmetry ratios (AR) were calculated from N1-P1 amplitudes using the following formula:


$$\begin{array}{l} AR=\;\frac{\left|left\;side\;N1-P1\;-\;right\;side\;N1-P1\right|}{\left|left\;side\;N1-P1\;\pm \;right\;side\;N1-P1\right|}\;x\;100 \end{array}$$

The ARs range from 100% to 0% with values near 0% indicating that N1-P1 amplitudes are symmetrical. The criterion for abnormal oVEMP was defined as an absent oVEMP at 142 dB _peak_FL and/or an amplitude asymmetry ratio ≥40% (laboratory normative data).

#### Balance and gait assessment

The Sensory Organization Test (SOT) was used to assess sensory integration for balance through measurement of postural sway under conditions in which visual and somatosensory feedback is systematically altered (32). The SOT is organized into a series of six conditions of increasing complexity and difficulty (Smart EquiTest, NeuroCom, Division of Natus, Pleasanton, CA, USA). The first three conditions were performed on a firm surface with eyes open, eyes closed and with vision sway referenced. The final three conditions were performed with the support surface sway-referenced with eyes open, eyes closed, and with vision sway-referenced. Sway-referencing refers to either the visual surround or support surface moving in the same direction and amplitude as the participant's sway which provides inaccurate visual or somatosensory input. Results of the SOT were calculated based on the theoretical maximum peak-to-peak anterior-posterior sway and expressed as an equilibrium score ranging from 0 to 100, with 0 indicating loss of balance and 100 indicating perfect stability. The composite equilibrium score (CES) was calculated as the weighted average of the equilibrium score for the six conditions. Abnormal SOT was defined as CES ≤70 for participants ages 20 to 59 years, and a CES ≤68 for participants 60 years or older (NeuroCom, 2011).

The Dynamic Gait Index (DGI) was used to assess balance during eight functional gait tasks that include walking on level ground, changing gait speed, walking with vertical and horizontal head turns, stepping over and around obstacles, and descending and ascending stairs (33). A trained clinician rated each walking task using a three-point ordinal scale with 0 indicating severe impairment and 3 indicating normal ability. The maximum total score (range 0 to 24) was calculated, and a score of ≤19 was considered abnormal (34).

Gait speed was measured with a stopwatch as participants walked at their normal preferred walking pace across 6 meters (m) on a level indoor surface without an assistive device. Three trials were performed, and the average speed was calculated for each participant. Participants began each trial 1.5 m from the starting point and continued walking for 1.5 m past the end point of the 6-m distance. Timing began when the first foot crossed the start point and ended when both feet crossed the end point. Participants wore a safety belt and were accompanied by a trained clinician to ensure safety. Abnormal gait speed was defined as <1.1 m/s as this cut-off has been linked to falls and other adverse events in older adults (35).

### Statistical analyses

All statistical analyses were performed using SPSS (version 28.0; SPSS Inc., Chicago, IL). Multiple analyses of variance (MANOVA) and *t*-tests were used for comparisons of vestibular and balance function between individuals with dizziness following mTBI/blast and a control group. Separate MANOVAs were conducted to determine the effect of group (control group, mTBI/blast group) on (1) saccadic accuracy (2 groups × 2 directions), (2) saccadic latency (2 groups × 2 directions), (3) saccadic velocity (2 groups × 2 directions), (4) smooth pursuit gain (2 groups × 3 frequencies), (5) SHA gain (2 groups × 3 frequencies), (6) SHA phase (2 groups × 3 frequencies), (7) cVEMP amplitude (2 groups × 2 sides), (8) cVEMP latency [2 groups × 2 sides × 2 peaks (P1 and N1)], (9) oVEMP amplitude (2 groups × 2 sides), and (10) oVEMP latency [2 groups × 2 sides × 2 peaks (N1 and P1)]. Separate univariate ANOVAs were performed for MANOVA outcome variables that showed a significant main effect. Absent cVEMP and oVEMP responses were assigned an amplitude value of 0 μV; absent responses were not included in the latency analyses. Pillai's Trace was used in each MANOVA to determine significance with *p* < 0.05 being considered statistically significant.

Independent-samples t-tests were conducted to determine the effect of group on caloric weakness, gain during visual fixation at 0.16 Hz, cVEMP asymmetry ratio, oVEMP asymmetry ratio, SOT composite score, DGI total score, and gait speed.

Vestibular, ocular motor, and balance test findings were categorized as normal or abnormal for each participant using established clinical cut-offs (published and laboratory normative data). These cut-offs are defined for each test described above and shown in each scatterplot figure for the test variables used to define a normal or abnormal test finding.

Chi-Square or Fisher's Exact tests were used to examine the association between mTBI/blast and abnormal vestibular and balance findings. Specifically, Fisher's Exact test was used when the frequency of any cell was <5, and Chi-Square was used in all other cases. Odds ratios were used to compare the odds of vestibular or balance dysfunction in individuals with dizziness associated with mTBI/blast exposure compared to healthy controls. It was hypothesized that mTBI/blast exposure produces negative outcomes in these measures, so one-sided *p*-values were used to determine significance at the 0.05 level.

## Results

### Participant characteristics

The demographics for each group are summarized in Table 1. Participants in the mTBI/blast group ranged in age from 22 to 67 years (mean = 38.1 years), and the control participants ranged in age from 20 to 60 years (mean = 31.6 years). Three participants in the mTBI/blast group and one in the control group were at least 60 years old. There was a significant difference in age between groups with the mean age for the control group ~8 years younger than the experimental group (p<0.001). Most participants in both groups were male (mTBI/blast: 96%; control: 88%).

**Table 1:** Group demographics and characteristics.

	**mTBI/Blast group**				**Control group**			
	**(*****N*** = **77)**				**(*****N*** = **32)**			
	***n* (%)**	**X**	**SD**	**Range**	***n* (%)**	**X**	**SD**	**Range**
Gender (male)	74 (96)				28 (88)			
Age (years)		38.1	10	22–67^*^		30.6	10	20–60^*^
DHI		48	23			0		
Onset (months)		96	90			n/a		
PTSD	62 (81)				n/a			
SNHL	56 (72)				7 (22)			
Mixed HL	2 (3)				0			

* Three participants in mTBI/blast group and one in control group are ≥60 years.

X, Mean; SD, Standard deviation; DHI, Dizziness Handicap Inventory; PTSD, Post-Traumatic Stress Disorder; SNHL, sensorineural hearing loss (one or more pure tone thresholds >25 dB HL in at least one ear); Mixed HL, Mixed hearing loss in at least one ear (one or more air conduction thresholds >25 dB HL and an air-bone gap >10 dB HL).

The mTBI/blast group was comprised of 52 individuals with a history of both mTBI and blast, 16 individuals with a history of blast only, and nine individuals with a history of mTBI only. The number of blast exposures reported by individual participants in the mTBI/blast group ranged from 1 to 50 (mean = 3.9). Fifty-one (75%) participants in the mTBI/blast group reported more than one blast exposure. Participants were queried about their distance from the blast when it detonated, and 34% indicated the blast was within five feet, 38% indicated 10 to 15 feet, and 25% indicated >15 feet. The time between study enrollment and the most severe blast exposure ranged from 6 to 564 months (mean = 95 months).

All participants in the mTBI/blast group reported dizziness following the mTBI and/or blast, and they were queried about their symptom characteristics. Imbalance and lightheadedness were the most common symptoms reported by 92 and 78% of participants, respectively. Both vertigo and lateropulsion were reported by 53% of participants, and oscillopsia was the least common symptom occurring in 23%. The Dizziness Handicap Inventory (DHI) was used as a measure of self-perceived balance handicap, and the mean DHI was 48 for the mTBI/blast group indicating moderate perceived handicap. Eighty-one percent of the mTBI/blast participants had a diagnosis of post-traumatic stress disorder.

**Figure 1:**
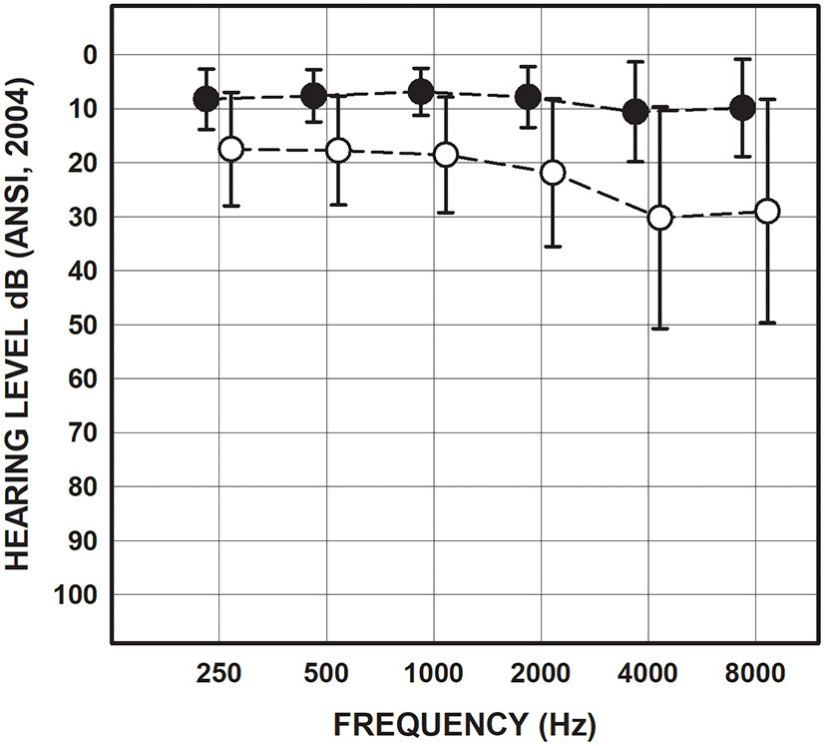
Mean and SDs for pure-tone thresholds collapsed across ears for the mTBI/blast (open circles) and control (closed circles) groups.

All participants had normal tympanometry bilaterally consistent with normal middle ear function. Pure tone audiometry revealed that 56 (72%) of the participants in the mTBI/blast group had sensorineural hearing loss in at least one ear and two had mixed hearing loss in one ear (Table 1). Seven participants (22%) in the control group had sensorineural hearing loss in at least one ear. Figure 1 shows the mean pure tone thresholds for the mTBI/blast and control groups. The mean pure tone average was 19.25 dB HL for the mTBI/blast group, and 7.4 dB HL for the control group (Figure 1).

### Vestibular function

The Dix-Hallpike and roll tests were negative for benign paroxysmal positional vertigo in all participants in both groups, thus no further analysis was performed.

#### Ocular motor

**Figure 2:**
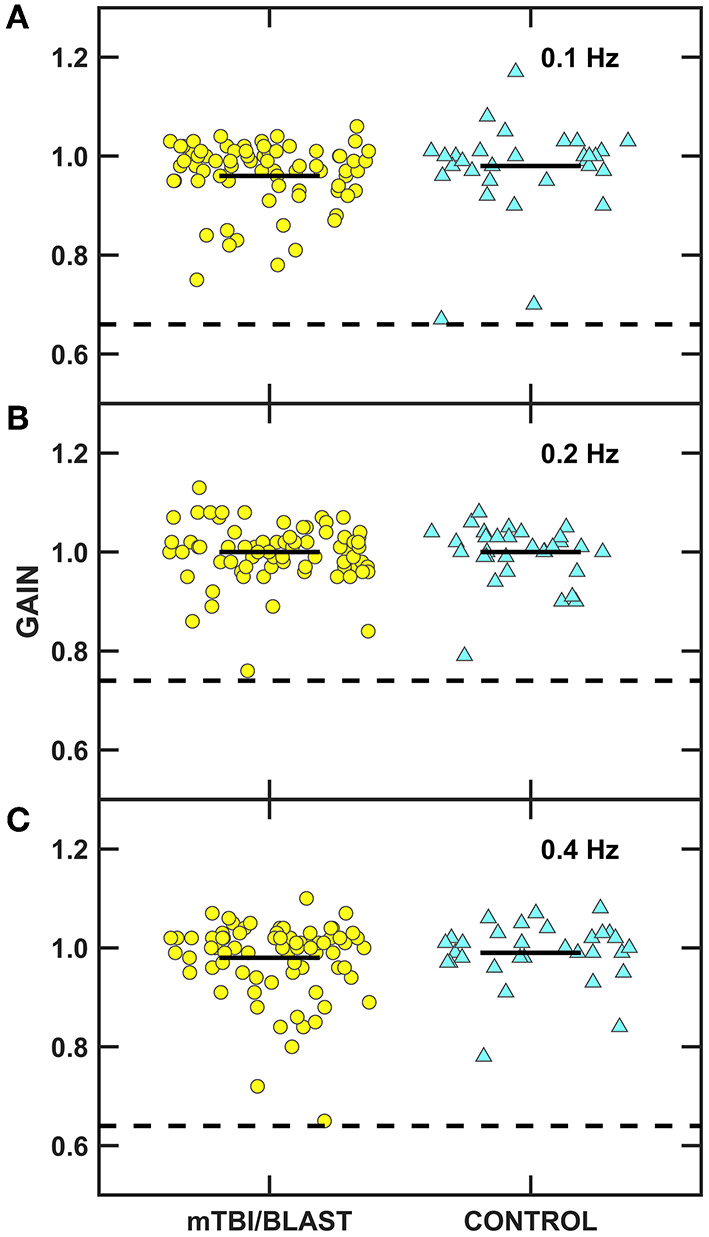
Scatterplots of gain for sinusoidal tracking at **(A)** 0.1 Hz, **(B)** 0.2 Hz, and **(C)** 0.4 Hz. Yellow circles represent individual data for participants with mTBI/blast, the blue triangles represent individual data for the healthy controls, and the black horizontal bars indicate group means. The dashed horizontal lines on each panel show the clinical cutoffs for gain at 0.1 Hz (<0.66), 0.2 Hz (<0.7), and 0.4 Hz (<0.64).

**Figure 3:**
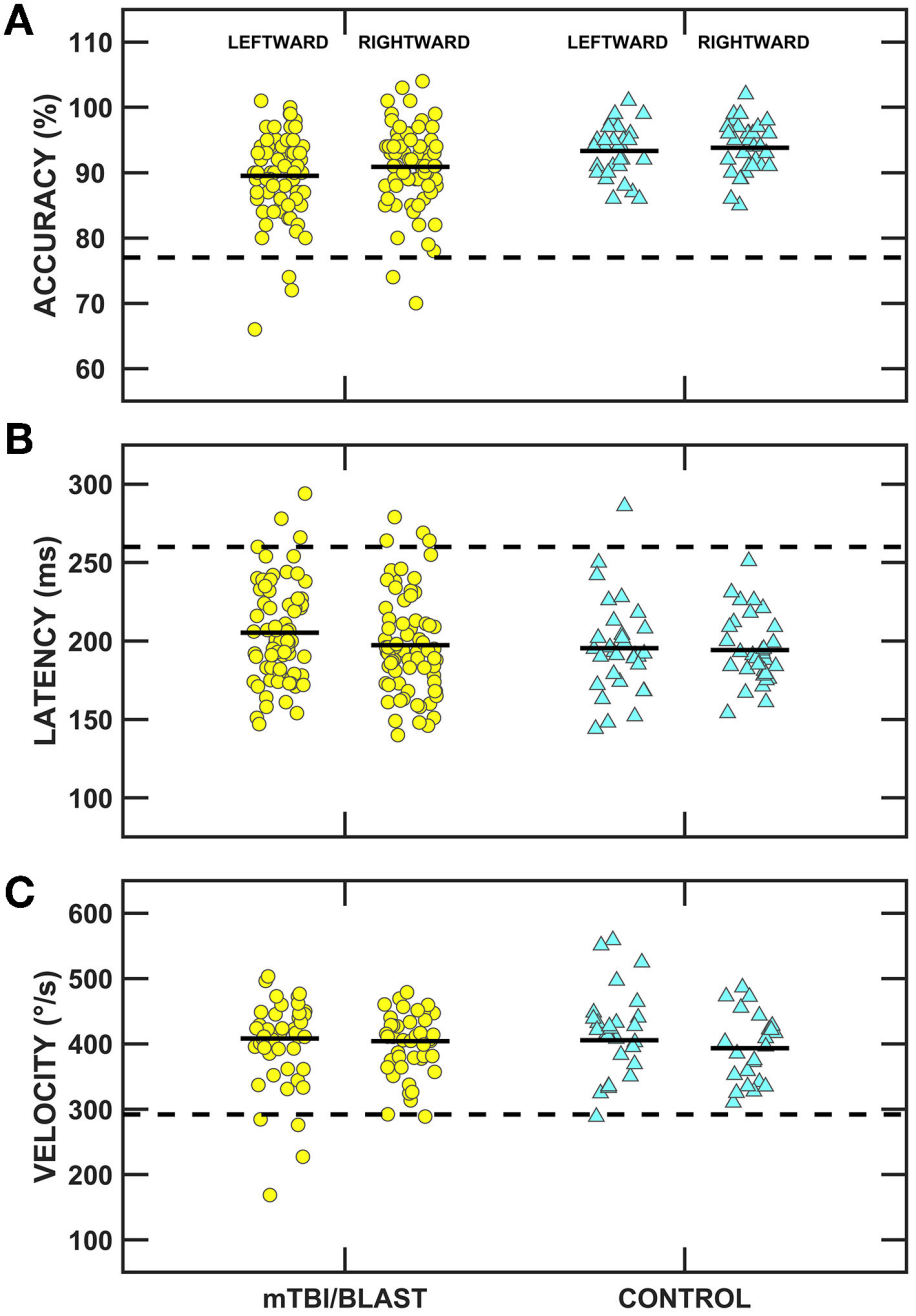
Scatterplots of accuracy **(A)**, latency **(B)**, and velocity **(C)** for leftward and rightward randomized saccades. Yellow circles represent individual data for participants with mTBI/blast, the blue triangles represent individual data for the healthy controls, and the black horizontal bars indicate group means. The dashed horizontal lines show the clinical cutoffs for: (panel A) mean accuracy (<77 or >137%) and (panel B) mean latency (>260 ms) for all saccadic eye displacements (5 to 25°), and (panel C) velocity for 18-25° saccadic eye displacements (<292°/s).

Ocular motor testing was used to assess central pathways. No participants in either group had gaze evoked nystagmus, and one participant in the mTBI/blast group had mild spontaneous nystagmus. Figure 2 shows individual and mean gain for sinusoidal smooth pursuit at 0.1, 0.2, and 0.4 Hz for both groups. There was no significant effect of group on smooth pursuit gain (*p* = 0.68), and all participants in both groups had normal smooth pursuit. Figure 3 shows individual and mean accuracy, latency, and velocity for leftward and rightward random saccades for both groups. A MANOVA indicated that saccadic accuracy was significantly poorer for the mTBI/blast group compared to the control group *(p* = 0.01; Table 2). Separate univariate ANOVAs revealed a significant effect of group on accuracy for both leftward [*F*_(1,101)_] = 9.03, *p* = 0.003 and rightward saccades [*F*_(1,101)_] = 5.88, *p* = 0.02. There were no significant group differences in saccadic latency (*p* = 0.21). Velocity data were excluded for 29 mTBI/blast participants and 8 controls due to inaccurate eye displacement (e.g., target undershoot) for 18 to 25° target displacement, and there was no significant group difference in saccadic velocity (*p* = 0.71). Fisher's exact test revealed no significant association between group and abnormal saccades (*p* = 0.11; Table 3).

**Table 2:** Means and standard deviations (SD) and group comparisons for vestibular function, ocular motor, balance, and gait measures by study group.

**Measures**	**mTBI/blast group (*****n*** = **77)**		**Control group (*****n*** = **32)**		***F***	***t***	***df***	^*^p
	**Mean**	**SD**	**Mean**	**SD**				
**Smooth pursuit**								
Gain					0.51		100	0.68
0.1	0.96	0.06	0.98	0.09				
0.2	1.00	0.06	1.00	0.06				
0.4	0.98	0.08	0.99	0.06				
**Saccades**								
Accuracy (%)					5.15		100	0.01
Left	89.51	6.56	93.32	3.90				0.003
Right	90.88	6.20	93.81	3.94				0.02
Latency (ms)					1.61		100	0.21
Left	205.29	31.18	195.42	30.21				
Right	197.32	31.53	194.23	22.05				
Velocity (deg/s)^**^					0.34		54	0.71
Left	408.23	69.93	405.56	50.71				
Right	404.11	46.97	393.36	48.55				
**Caloric**								
Unilateral weakness (%)	10.37	11.82	7.07	6.04		1.45	104	0.08
**Rotary chair**								
Gain					3.53		105	0.02
0.01	0.39	0.09	0.45	0.07				0.001
0.04	0.51	0.11	0.55	0.08				0.07
0.16	0.57	0.12	0.60	0.13				0.15
Phase					0.64		102	0.59
0.01	41.19	8.04	42.22	8.33				
0.04	11.28	6.15	12.91	4.28				
0.16	−1.93	11.64	−0.78	4.58				
**VFx**	0.02	0.05	0.02	0.04		0.20	102	0.42
**cVEMP**								
Amplitude (μV)					3.02		105	0.05
Left	1.61	1.46	1.63	1.22				
Right	1.37	1.2	1.9	1.63				
Latency (ms)					0.72		76	0.58
Left P1	15.75	1.98	15.97	2.31				
Right P1	15.15	1.42	15.72	1.99				
Left N1	23.85	2.37	24.28	2.12				
Right N1	24.06	2.35	24.34	2.45				
AR (%)	29	29.06	20	15.87		2.023	102	0.02
**oVEMP**								
Amplitude (μV)					0.31		106	0.73
Right	7.02	6.58	7.94	5.78				
Left	8.80	7.00	9.95	7.03				
Latency (ms)					1.20		91	0.32
Left N1	10.76	1.53	10.14	1.12				
Right N1	10.67	1.42	10.24	1.22				
Left P1	15.58	1.99	14.79	1.50				
Right P1	15.34	1.99	14.69	2.04				
AR (%)	18.6	14.57	17.5	14.69		0.33	103	0.37
**SOT**	63.9	18.4	80.7	5.1		7.06	99	<0.001
**DGI**	21.7	2.5	24	0.0		6.55	75	<0.001
**Gait speed (m/s)**	1.04	0.15	1.19	0.21		3.43	75	<0.001

* p-values for t-tests are one-tailed;

** 18 to 25° eye deflections.

AR, Amplitude asymmetry ratio; VFx, Visual fixation suppression; cVEMP, cervical vestibular evoked myogenic potential; oVEMP, ocular vestibular evoked myogenic potential; SOT, Sensory Organization Test; DGI, Dynamic Gait Index.

F-values are reported for MANOVA tests and t-values are reported for t-tests.

**Table 3:** Association between group and abnormal vestibular and balance test findings using Fisher's Exact test.

	**Odds ratio**	**95% Confidence interval**		^*^ *p*
		**Lower**	**Upper**	
**Vestibular**				
Caloric	1.4	1.25	1.62	0.18
SHA	1.5	1.27	1.65	0.12
VFx	3.0	0.35	25.67	0.27
cVEMP	3.4	0.93	12.36	0.04
oVEMP	2.1	0.57	8.06	0.20
**Balance**				
SOT	33.4	4.31	258.7	<0.001
DGI	1.5	1.28	1.79	0.03
Gait speed	5.4	1.88	15.72	^**^0.001

* One-tailed p-values;

** p-value is for Chi-Square test.

SHA, sinusoidal harmonic acceleration at 0.01 Hz; VFx, Visual fixation suppression; cVEMP, cervical vestibular evoked myogenic potential; oVEMP, ocular vestibular evoked myogenic potential; SOT, sensory organization test; DGI, dynamic gait index.

#### Horizontal semicircular canal/vor pathway

**Figure 4:**
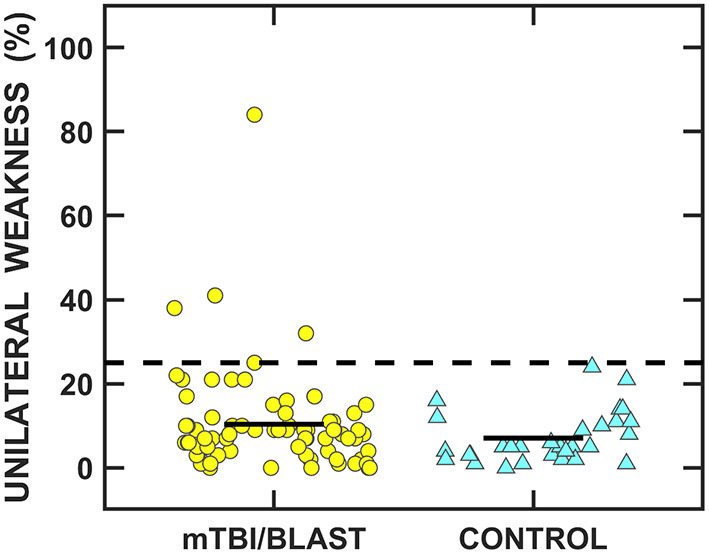
Scatterplots of unilateral weakness for the caloric test. Yellow circles represent individual data for participants with mTBI/blast, the blue triangles represent individual data for the healthy controls, and the black horizontal bars indicate group means. The dashed line shows the clinical cutoff for abnormal caloric weakness (>25%).

Caloric and rotational tests were used to determine the impact of mTBI/blast on the horizontal semicircular canals and the VOR pathways. Figure 4 shows individual and mean data for caloric weakness for both groups. The mean caloric weakness was 7% for the control group and 10% for the mTBI/blast group, and there was no significant effect of group (*p* = 0.08; Table 2). The dashed horizontal line in Figure 4 indicates the clinical cutoff for abnormal caloric weakness (>25%), and five individuals in the mTBI/blast group and none of the controls demonstrated an abnormal caloric test finding. Fisher's exact test revealed no significant association between group and caloric weakness (*p* = 0.18; Table 3).

**Figure 5:**
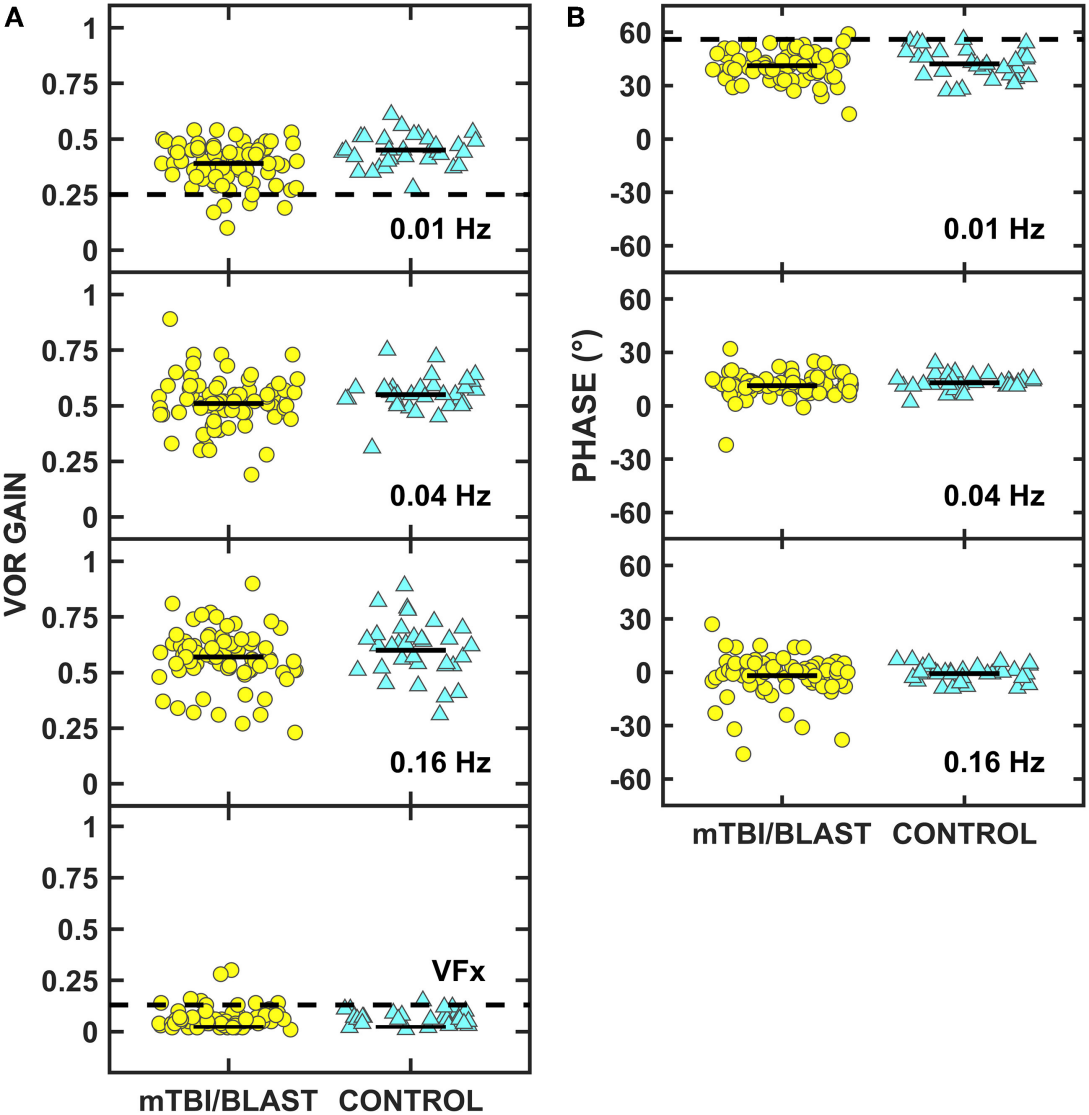
Scatterplots of VOR gain **(A)** and phase **(B)** for the rotary chair slow harmonic acceleration test. Yellow circles represent individual data for participants with mTBI/blast, the blue triangles represent individual data for the healthy controls. The dashed horizontal lines on panel A show the clinical cutoffs for VOR gain at 0.01 Hz (<0.25) and during visual fixation at 0.16 Hz (>0.13), and the dashed line on panel B shows the cutoff for phase at 0.01 Hz (>56).

Figure 5A shows individual and mean data for rotary chair SHA gain at 0.01, 0.04, and 0.16 Hz and during visual fixation at 0.16 Hz for both groups. A MANOVA revealed a significant main effect of group on gain (*p* = 0.02; Table 2) and separate univariate ANOVAs at 0.01, 0.04, and 0.16 Hz revealed a significant effect of group on gain only at 0.01 Hz, [*F*_(1,107)_] = 10.68, *p* = 0.001; the gain at 0.01 Hz for the mTBI/blast group was significantly lower than the gain of the control group. There was no significant effect of group on gain for visual fixation suppression (*p* = 0.42; Table 2). Individual and mean data for phase across rotary chair SHA frequencies are shown in Figure 5B and there was no significant effect of group on VOR phase (*p* = 0.59). The dashed horizontal lines in Figures 5A,B indicate clinical cutoffs for normal gain and phase at 0.01 Hz. Six individuals in the mTBI/blast group and no control participants met the clinical cutoff criteria for abnormal VOR gain at 0.01 Hz. Fisher's exact test revealed no significant association between group and clinically abnormal rotary chair findings (*p* = 0.18; Table 3).

#### Otolith organs

**Figure 6:**
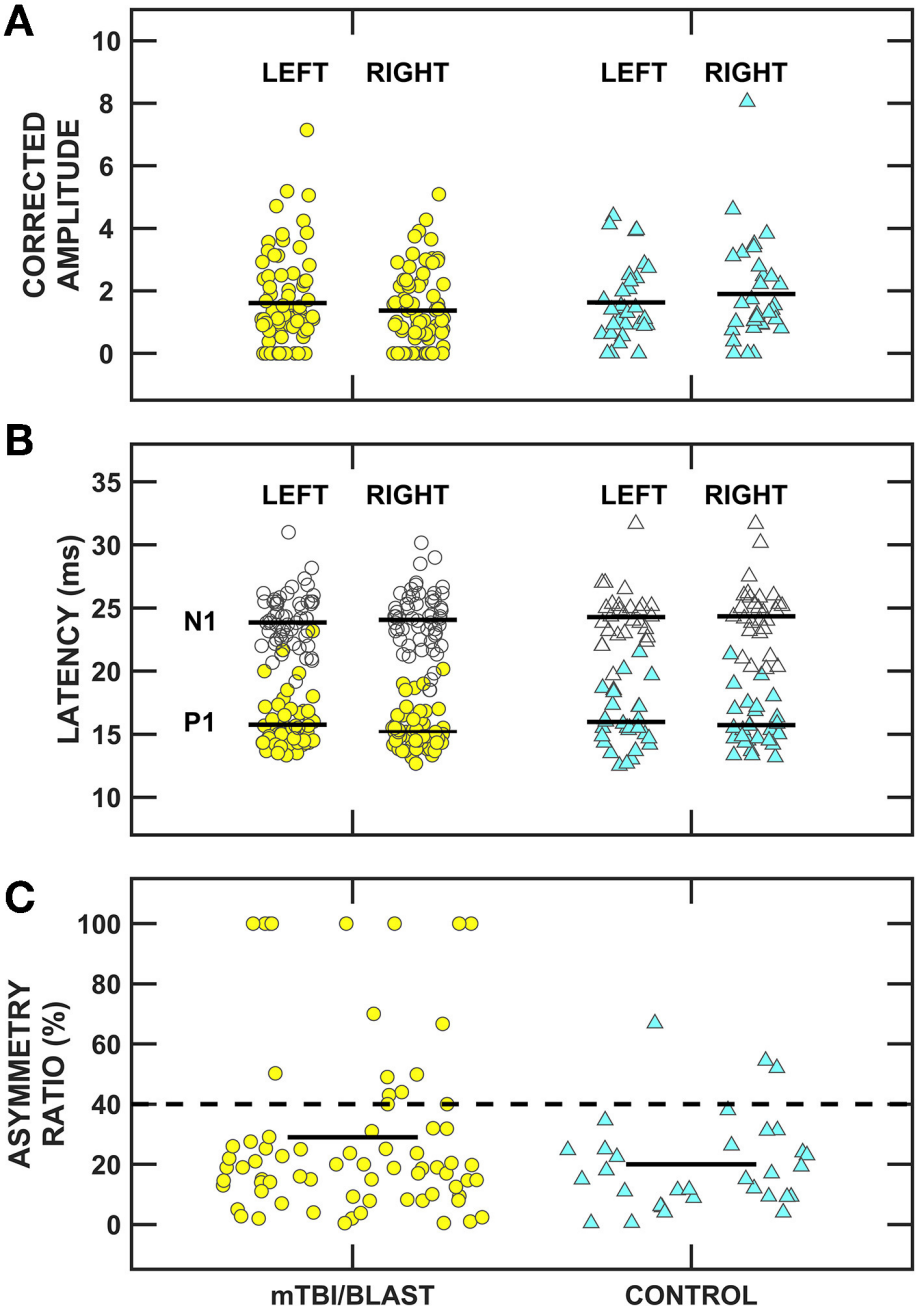
Scatterplots of **(A)** corrected P1/N1 amplitude, **(B)** P1 (filled symbols) and N1 (open symbols) latency, and **(C)** inter-ear amplitude asymmetry ratio for cervical vestibular evoked myogenic potentials (cVEMPs). Yellow circles represent individual data for participants with mTBI/blast, the blue triangles represent individual data for the healthy controls, and the black horizontal bars indicate group means. The dashed horizontal line in **(C)** shows the clinical cutoff for abnormal asymmetry ratio (>40%).

The impact of mTBI/blast on the otolith organs and their pathways was assessed using cervical and ocular VEMPs. Figure 6 shows individual and mean cVEMP data for amplitude (panel A), latency (panel B) and asymmetry ratio (panel C) for the mTBI/blast and control groups. A MANOVA revealed that there was no significant main effect of group on cVEMP amplitude (*p* = 0.05) or latency (*p* = 0.58). In contrast, the mTBI/blast group had a significantly greater asymmetry ratio than the control group (*p* = 0.02; Table 2). The dashed horizontal line in Figure 6C indicates the clinical cutoff (>40%) for abnormal cVEMP asymmetry ratio. Nineteen individuals in the mTBI/blast group and two control participants demonstrated an abnormal cVEMP. Fisher's exact test revealed a significant association between group and abnormal cVEMP findings (*p* = 0.04; Table 3); the participants in the mTBI/blast group were 3.4 times more likely to have an abnormal asymmetry ratio than participants in the control group.

**Figure 7:**
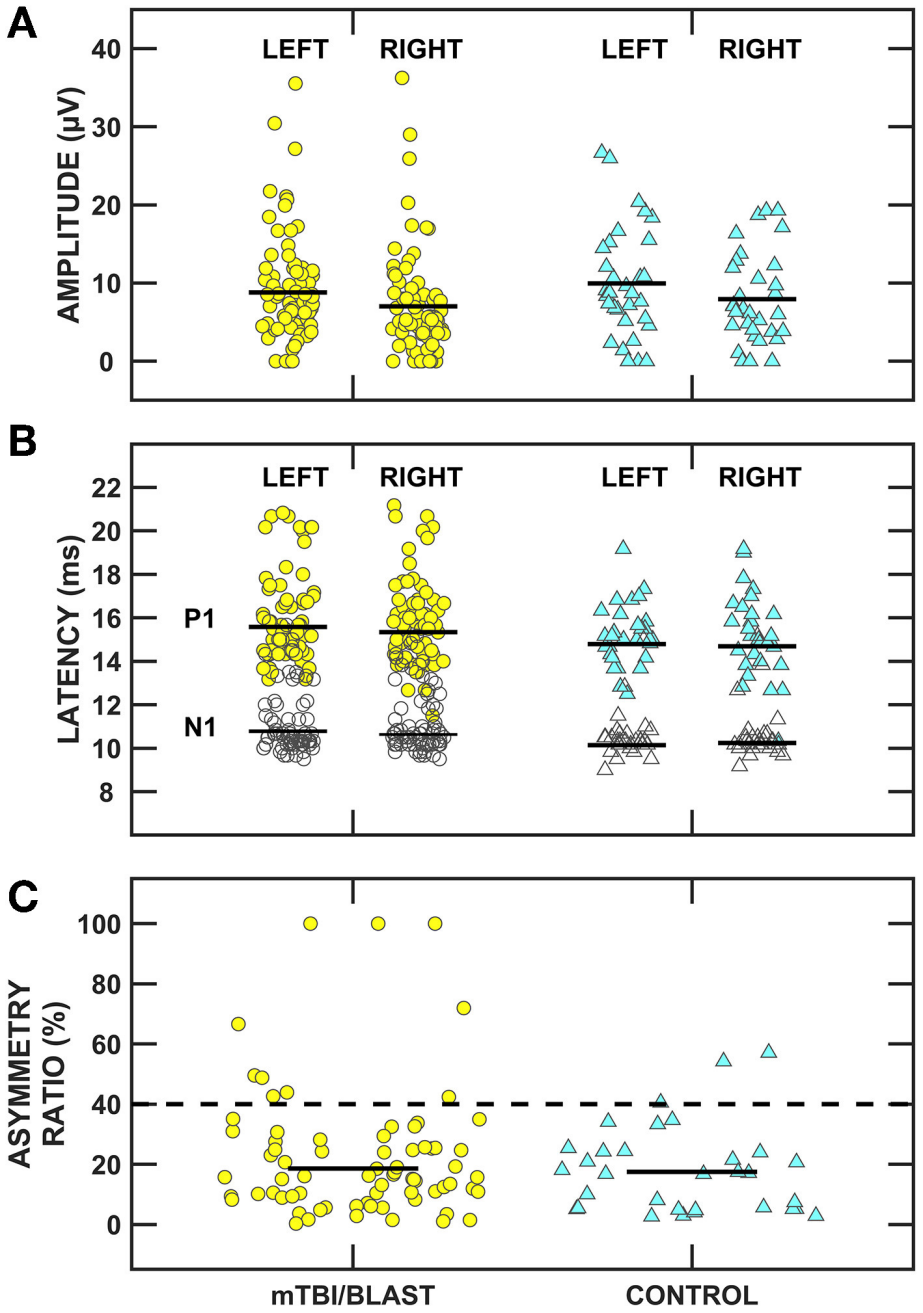
Scatterplots of **(A)** N1/P1 amplitude, **(B)** N1 (filled symbols) and P1 (open symbols) latency, and **(C)** inter-ear amplitude asymmetry ratio for ocular vestibular evoked myogenic potentials (oVEMPs). Yellow circles represent individual data for participants with mTBI/blast, the blue triangles represent individual data for the healthy controls, and the black horizontal bars indicate group means. The dashed horizontal line in **(C)** shows the clinical cutoff for abnormal asymmetry ratio (>40%).

Figure 7 shows individual and mean oVEMP data for amplitude (panel A), latency (panel B) and asymmetry ratio (panel C) of the mTBI/blast and control groups. There was no significant main effect of group on amplitude (*p* = 0.73), latency (*p* = *0*.32), or asymmetry ratio (*p* = 0.37) (Table 2). The dashed horizontal line in panel C indicates the clinical cutoff (>40%) for abnormal oVEMP asymmetry ratio. Fourteen individuals in the mTBI/blast group and three control participants demonstrated an abnormal oVEMP. Fisher's exact test revealed no significant association between group and abnormal oVEMP findings (*p* = 0.20; Table 3).

### Balance and gait function

**Figure 8:**
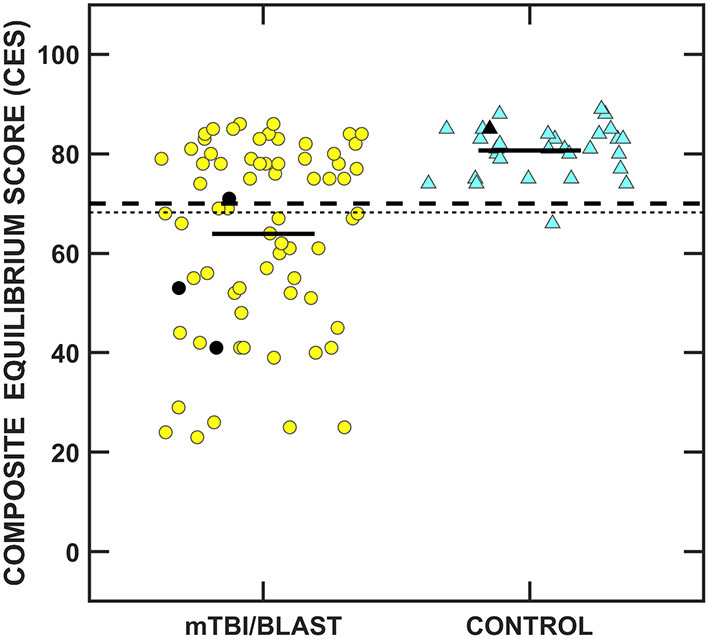
Scatterplots of the composite equilibrium score (CES) for the Sensory Organization Test. Yellow circles represent individual data for participants with mTBI/blast, the blue triangles represent individual data for the healthy controls, and the black horizontal bars indicate group means. Individual data for participants >60 years of age are indicated by black-filled symbols. The dashed line indicates the clinical cutoff for 20- to 59-year-old participants for abnormal CES (≤70), and the dotted line indicates the clinical cutoff for participants >60 years of age for abnormal CES (≤68).

Figure 8 shows individual and mean SOT CES for the mTBI/blast and control groups. There was a significant effect of group on the SOT CES (*p* ≤ 0.001; Table 2) and the mean CES scores was 80.7 for the control group and 63.9 for the mTBI/blast group. The dashed horizontal line in Figure 8 indicates the clinical cutoff for 20- to 59-year-old participants. Thirty-eight individuals in the mTBI/blast group and one control participant demonstrated abnormal SOT (CES). Fisher's exact test revealed a significant association between group and abnormal SOT findings (*p* ≤ 0.001; Table 3); the participants in the mTBI/blast group were 33 times more likely to have an abnormal SOT CES than participants in the control group.

**Figure 9:**
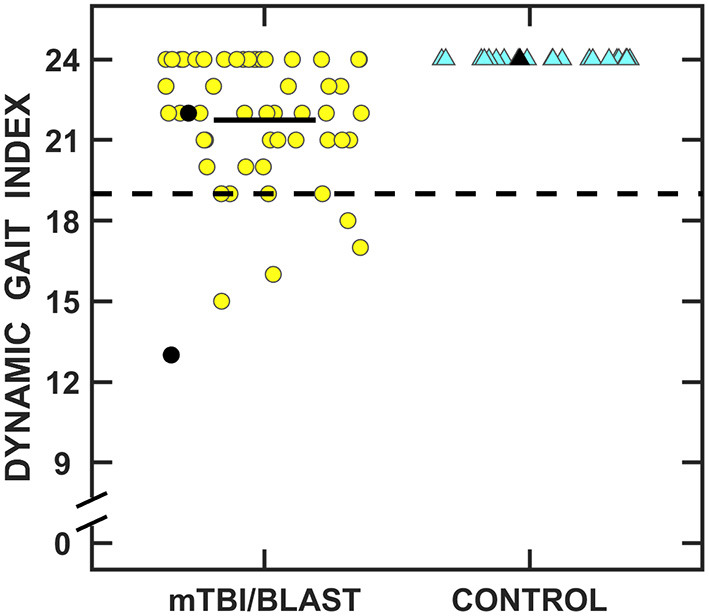
Scatterplots of the total Dynamic Gait Index scores. Yellow circles represent individual data for participants with mTBI/blast, and the blue triangles represent individual data for the healthy controls. Individual data for participants >60 years of age are indicated by black-filled symbols. The black horizontal bar indicates the mTBI/blast group mean. The dashed line shows the clinical cutoff for abnormal DGI (<19).

Figure 9 shows individual and mean total Dynamic Gait Index scores for the mTBI/blast and control groups. The mean scores were 24 for the control group and 21.7 for the mTBI/blast group, and there was a significant effect of group on DGI (*p* ≤ 0.001; Table 2). The dashed horizontal line in Figure 9 indicates the clinical cutoff for normal DGI. Nine individuals in the mTBI/blast group and none of the control participants demonstrated an abnormal DGI. Chi-Square revealed a significant association between group and abnormal DGI findings (*p* = 0.03; Table 3); the participants in the mTBI/blast group were 7.5 times more likely to have an abnormal total DGI than participants in the control group.

**Figure 10:**
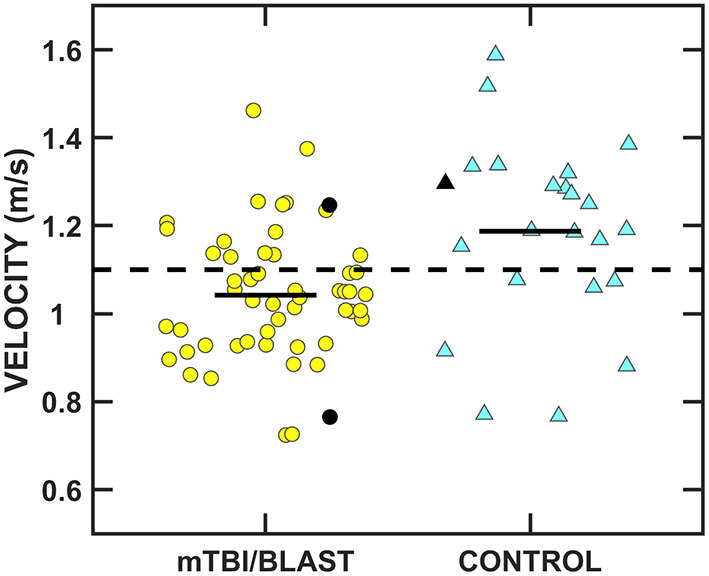
Scatterplots of mean gait speed. Yellow circles represent individual data for participants with mTBI/blast, the blue triangles represent individual data for the healthy controls, and the black horizontal bars indicate group means. Individual data for participants >60 years of age are indicated by black-filled symbols. The dashed line shows the clinical cutoff for abnormal gait speed (<1.1 m/s).

Figure 10 shows individual and mean gait speed for the mTBI/blast and control groups. The mean speed was 1.19 m/s for the control group and 1.04 m/s for the mTBI/blast group, and there was a significant effect of group on gait speed (*p* ≤ 0.001; Table 2). The dashed horizontal line in Figure 10C indicates the clinical cutoff used to categorize participants with normal/abnormal gait speed. Thirty-eight individuals in the mTBI/blast group and 7 control participants demonstrated abnormal gait speed. Fisher's exact test revealed a significant association between group and abnormal gait speed (*p* = 0.001; Table 3); the participants in the mTBI/blast group were 5.4 times more likely to have an abnormal gait speed than participants in the control group.

## Discussion

**Figure 11:**
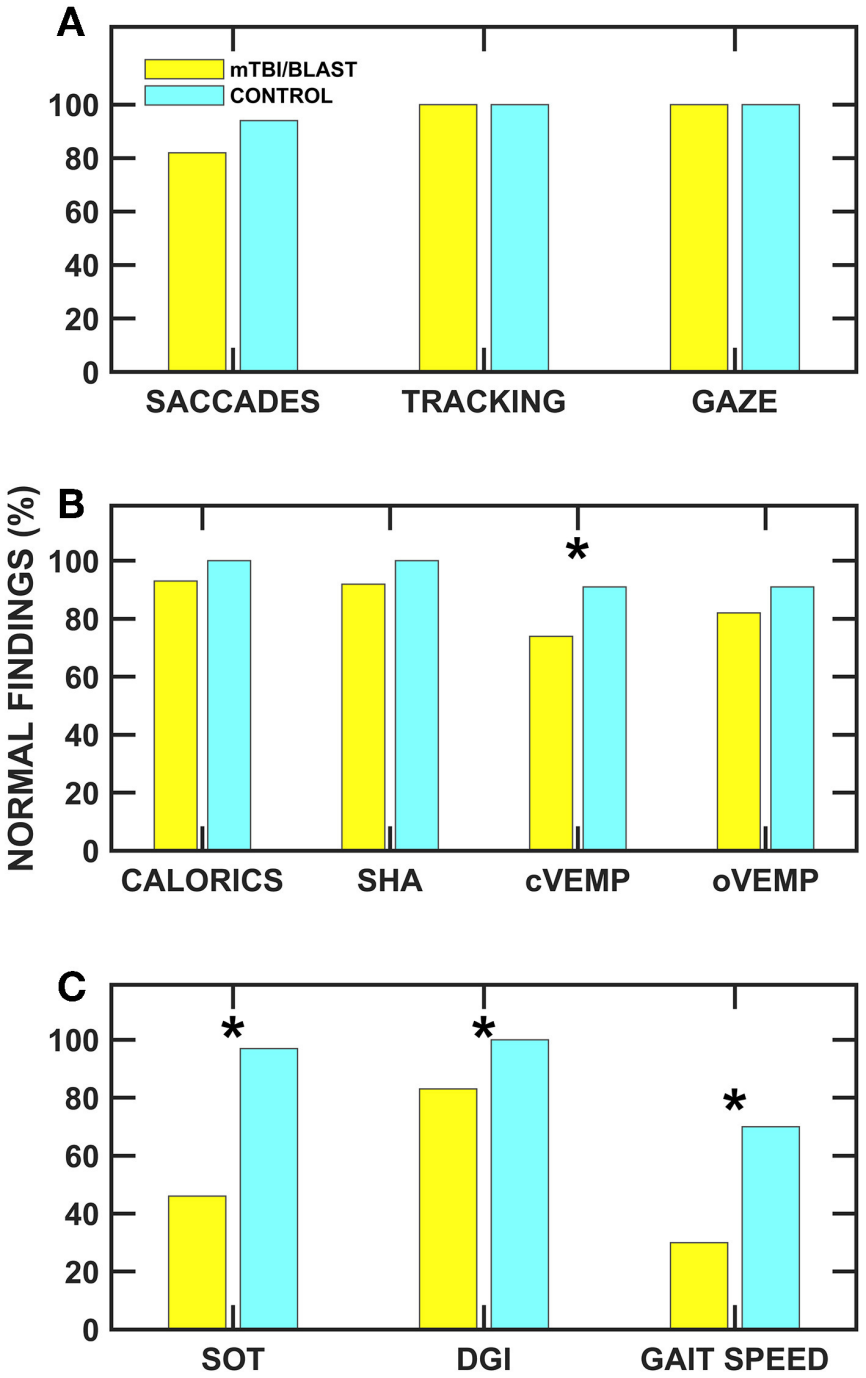
Frequency of normal ocular motor **(A)**, vestibular **(B)**, and balance **(C)** findings for the mTBI/blast (blue) and control (yellow) groups. The asterisks (*) indicate significant associations (*p* < 0.05) between group and abnormal test findings.

Individuals with mTBI/blast and chronic dizziness demonstrated decreased vestibular and balance function compared to healthy controls. The frequency of test abnormalities in participants with mTBI/blast ranged from 0 to 70% across vestibular, ocular motor, and balance testing, with the most frequent abnormalities occurring on tests of balance and gait function (Figure 11). Seventy-two percent of the mTBI/blast participants (*n* = 56) had abnormal findings on one or more of the balance and gait tests, and there were significant group differences for all measures of balance and gait. The mean SOT (63.9) for the mTBI/blast group was abnormal (<68), and individuals with mTBI/blast and dizziness were 33 times more likely to have abnormal postural instability on SOT than healthy controls. The SOT assesses the integration of sensory information for static balance by measuring postural sway under conditions in which visual and somatosensory feedback is altered. Our findings are consistent with numerous studies that have examined postural stability using SOT and demonstrated higher magnitudes of anterior-posterior sway in individuals with mTBI than healthy controls [e.g., (36)]. Using a mouse model, Lien and Dickman (37) observed a significant reduction in the ability to perform the righting reflex and balance on a rotating rod that lasted several weeks post-blast exposure. In addition to abnormal static postural instability, the mTBI/blast group performed poorer on gait tasks compared to the healthy control group, and these findings are consistent with other studies that examined the impact of mTBI on balance in humans (38, 39). Overall, the mTBI/blast group walked slower than the healthy control group and their average gait speed has been associated with adverse events, including hospitalization and falls (35). Although there was a ceiling effect for DGI in the control group, individuals in the mTBI/blast group performed poorer than controls suggesting reduced dynamic equilibrium including changing walking speed, walking with head turns and pivot turns.

Vestibular test abnormalities occurred in ~34% of the individuals with chronic dizziness and mTBI/blast, and abnormalities occurred more frequently for measures of otolith organ function (25% for cVEMP and 18% for oVEMP) than for measures of hSCC function (8% for SHA and 6% for caloric test). Although there were significant group differences for measures of cVEMP and SHA (0.01 Hz), the association between mTBI/blast and abnormal findings was only significant for cVEMP. The odds ratio suggested that individuals with mTBI/blast and dizziness were 3.4 times more likely to have abnormal cVEMPs (absent response or >40% AR) than healthy controls. These findings are consistent with previous studies and suggest the sacculo-collic pathway may be susceptible to damage from both mTBI and blast exposure. Serrador et al. (40) used unilateral centrifugation to assess otolith function in a group of Veterans with blast and/or mTBI and found that 30% demonstrated unilateral otolith dysfunction without horizontal canal impairments. Ernst and colleagues (12) demonstrated otolith disorders in 25% of patients who experienced a blunt force head trauma. Recently, Gard et al. (41) reported abnormal cVEMPs in 38% of athletes with persistent post-concussive symptoms compared to no cVEMP abnormalities in a control group. A histological study in humans support the abnormal otolith findings in our cohort and suggests that the saccule (one of the otolith organs) may be particularly susceptible to blast-related damage owing to the anatomic proximity of the saccule to the stapes footplate (13). In contrast to the current and previous studies that have shown significant effect of mTBI/blast on vestibular function, Modica et al. (42) recently showed no significant differences for vestibular or ocular motor outcome measures in a small group (*n* = 20) of career breachers (individuals that utilize explosives) vs. non-breachers.

Several recent animal studies have also demonstrated blast exposure-induced damage to the vestibular receptors and afferents as well as reductions in balance function. Lien and Dickman (37) exposed mice to a 63 kPa peak blast-wave over pressure and observed the following post-exposure changes: (1) significant loss of hair cell stereocilia in the cristae and macule up to one-month post-exposure, and (2) significant reduction in horizontal VOR gain and phase lags that lasted many weeks following a single blast exposure event. Yu et al. (43) exposed anesthetized rats to blast shock waves (~20 PSI) delivered to the external canal and observed a significant reduction in the spontaneous discharge rates of the otolith and canal afferents and a reduction in the sensitivity of irregular canal afferents to sinusoidal head rotation at 0.5 to 2 Hz. In contrast to the findings of Lien and Dickman (37), Yu et al. (43), observed few changes in the VOR responses to sinusoidal head rotation and translation.

Ocular motor testing abnormalities occurred in 18% of the individuals with mTBI/blast. No gaze-evoked nystagmus was present and smooth pursuit was normal in all individuals with mTBI/blast. Saccadic velocity and latency were similar for the mTBI/blast and control groups; however, there was a significant group difference in saccadic accuracy (13 individuals with mTBI/blast had abnormal saccadic accuracy). Ocular motor testing can be used to screen central nervous system (CNS) function independent of peripheral vestibular system function, and abnormal ocular motor function (e.g., saccadic dysmetria, gaze-evoked nystagmus, or saccadic pursuit) can suggest central abnormalities across diverse neurological pathways that include the cerebral hemispheres, cerebellum, and brainstem. Previous studies have reported gaze evoked nystagmus, and break-up of smooth pursuit in individuals with mTBI and blast exposure although the frequency of abnormal findings is low [e.g., (5, 44)]. In contrast, Scherer et al. (19), reported a relatively higher incidence (45%) of ocular motor dysfunction in a group of symptomatic and asymptomatic service members recovering from blast-related TBI. The disparity in findings may in part be explained by differences in time since onset of injury, injury severity, and definitions of abnormality which often differ between studies. Recently, Cochrane et al. (45) observed increased saccadic latency and decreased accuracy in athletes who suffered concussions compared to non-concussed athletes, but there was no group difference in horizontal smooth pursuit. Convergence insufficiency has been associated with blast-related TBI [e.g., (46)]; however, versional eye movement was not assessed in the current study. A limitation of ocular motor tests for detecting CNS pathology is the use of prescribed medications that impact ocular motor function (47). In the current study, we requested that participants refrain from use of alcohol, recreational drugs, over-the-counter antihistamines, anti-dizzy medications, and sleeping pills 48 h prior to testing.

Central vestibular function was assessed using visual fixation (Vfx) suppression during slow harmonic acceleration at 0.16 Hz. We observed no significant effect of group on gain for Vfx suppression, and 10% of the participants with mTBI had abnormal VFx gain. Fixation suppression of vestibular nystagmus requires intact connections between the cerebellum and vestibular nuclei and has been used to assess central vestibular involvement or visual-vestibular interaction.

There are several limitations of this study that should be considered. First, the heterogeneity of the type of injury in the experimental group limits the specificity of the findings to mTBI or blast injury. Due to the small sample size of individuals with mTBI only, we combined individuals with mTBI only, blast only, and mTBI and blast into one experimental group rather than three separate groups. In a systematic review of outcomes associated with blast vs. non-blast related TBI in US military service members and Veterans, Greer et al. (48) concluded that most clinical and functional outcomes (including vestibular dysfunction) appeared comparable in military service members and Veterans with TBI, regardless of blast exposure but indicated that the data were limited and that more research was needed to determine whether there is a distinct pattern of impairments and comorbidities associated with blast-related TBI. Denby et al. (8) reported that the likelihood of vestibular disturbance is influenced by the way mTBI was acquired. Specifically, they observed that 83% of participants with blunt + blast mTBIs reported a vestibular disturbance compared to participants with either blunt mTBI (59%) or blast mTBI (47%). The authors noted that the vestibular results were based on self-report data (Vertigo Symptom Scale Long form), rather than prospective clinical examination, which may have led to an overestimation of effect.

Another limitation of the study is the statistically significant age difference between the mTBI/blast and control groups (mean age was 38.1 years for mTBI/blast group and 30.6 for control group). Most participants in each group were relatively young and most age-related changes to the vestibular and balance systems occur past the fourth decade [e.g, (49)]. The scatterplots show individual data highlighted for three participants in the mTBI/blast group and one participant in the control group who were >60 years of age.

In the current study, the assessment of hSCC/VOR function was limited to caloric and rotational tests. These tests assess VOR function at frequencies ranging from 0.003 to 0.64 Hz, whereas the frequencies of natural head movement occur at ~1–5 Hz (50). The video head impulse test (vHIT) has been increasingly used to assess the gain of the high-frequency VOR for the horizontal and vertical SCCs. A recent study revealed normal VOR gain for horizontal and vertical SCC vHIT in a group of age-matched non-TBI veterans (*n* = 45) and a group of veterans (*n* = 25) with chronic mild or moderate TBI (51). Overt and covert corrective saccades were recorded in ~25% of the head impulses within both groups of veterans. The clinical significance of these findings is unclear as corrective saccades have been observed also in healthy older adults with normal VOR gain (52).

Finally, there was a high incidence (81%) of PTSD in the experimental group. For many veterans of the wars in Iraq and Afghanistan, a history of TBI is associated with exposure to a traumatic event (e.g., blast exposure) that increases the risk of PTSD (53). Although PTSD was not assessed in the control group, it is likely that fewer controls had PTSD as they did not have a history of mTBI or blast and were less likely to have served in combat. There is evidence that balance and gait impairments may be associated with increased incidence of PTSD [e.g., (54)]. Thus, it is difficult to rule out the role of PTSD on the balance and gait findings in this study. Most studies that have examined the effect mTBI/blast on vestibular function have used subjective measurements such as questionnaires or the presence of symptoms or screening tests to assess vestibular function [e.g., (8, 55)]. In contrast, the present study used quantitative laboratory tests to determine the effects of TBI and/or blast exposure on vestibular function thereby reducing the influence of PTSD on the vestibular outcomes.

Clinical guidelines provide recommendations on treatment approaches specifically for dizziness related to mTBI (56, 57). A trial of vestibular rehabilitation and balance therapeutic (VRT) exercise provided by specialty trained therapists is recommended for chronic dizziness and imbalance resulting from mTBI (57); however, the strength of the evidence for VRT was weak to moderate based on a limited number of randomized controlled trials (56, 57). The general approach to patients with dizziness related to mTBI is problem-based, involving customized exercises and progressions to address the identified impairments and limitations (58). A retrospective chart review of 114 patients with mTBI/concussion referred for VRT home exercise plans revealed that the most frequently prescribed exercise category was eye-head coordination exercises (95%) followed by static balance (88%) and gait (76%) training (59). The results of the current study support the inclusion of balance and gait training as an important component of treatment for patients with dizziness related to mTBI/blast.

In summary, these findings suggest that mTBI/blast more often impacts postural stability than vestibular function. Vestibular abnormalities occurred more frequently for measures of saccular-collic pathway function (cVEMP) than for measures of hSCC function. These findings support the need for comprehensive vestibular and balance assessment in individuals with dizziness following mTBI/blast-related injury.

## Data availability statement

The raw data supporting the conclusions of this article will be made available by the authors, without undue reservation.

## Ethics statement

The studies involving human participants were reviewed and approved by ETSU/VA Medical IRB. The patients/participants provided their written informed consent to participate in this study.

## Author contributions

FA and OM were responsible for funding and oversaw data acquisition, contributed to conception, and design of the study. FA wrote the first draft of the manuscript. All authors contributed to manuscript revision, read, and approved the submitted version.

## Funding

This material is based upon work supported by the U.S. Department of Veterans Affairs, Rehabilitation Research and Development under Award No. IRX000189A.

## Conflict of interest

The authors declare that the research was conducted in the absence of any commercial or financial relationships that could be construed as a potential conflict of interest. The reviewers BH and AM declared a shared affiliation, though no other collaboration, with one of the authors KR at the time of the review.

## Publisher's note

All claims expressed in this article are solely those of the authors and do not necessarily represent those of their affiliated organizations, or those of the publisher, the editors and the reviewers. Any product that may be evaluated in this article, or claim that may be made by its manufacturer, is not guaranteed or endorsed by the publisher.

## Author disclaimer

The views, opinions, and/or findings expressed in this publication are those of the authors and should not be construed as an official Department of Veterans Affairs position, policy, or decision.
